# Activation of the Low Molecular Weight Protein Tyrosine Phosphatase in Keratinocytes Exposed to Hyperosmotic Stress

**DOI:** 10.1371/journal.pone.0119020

**Published:** 2015-03-17

**Authors:** Rodrigo A. Silva, Marcelly V. Palladino, Renan P. Cavalheiro, Daisy Machado, Bread L. G. Cruz, Edgar J. Paredes-Gamero, Maria C. C. Gomes-Marcondes, Willian F. Zambuzzi, Luciana Vasques, Helena B. Nader, Ana Carolina S. Souza, Giselle Z. Justo

**Affiliations:** 1 Departamento de Bioquímica, Instituto de Biologia, Universidade Estadual de Campinas, Campinas, São Paulo, Brazil; 2 Departamento de Bioquímica (Campus São Paulo), Universidade Federal de São Paulo, São Paulo, São Paulo, Brazil; 3 Departamento de Química e Bioquímica, IBB, Universidade Estadual Paulista, Botucatu, São Paulo, Brazil; 4 Departamento de Genética e Biologia Evolutiva, Universidade de São Paulo, São Paulo, São Paulo, Brazil; 5 Centro de Ciências Naturais e Humanas, Universidade Federal do ABC, Santo André, São Paulo, Brazil; 6 Departamento de Bioquímica (Campus São Paulo) and Departamento de Ciências Biológicas (Campus Diadema), Universidade Federal de São Paulo, São Paulo, São Paulo, Brazil; Casey Eye Institute, UNITED STATES

## Abstract

Herein, we provide new contribution to the mechanisms involved in keratinocytes response to hyperosmotic shock showing, for the first time, the participation of Low Molecular Weight Protein Tyrosine Phosphatase (LMWPTP) activity in this event. We reported that sorbitol-induced osmotic stress mediates alterations in the phosphorylation of pivotal cytoskeletal proteins, particularly Src and cofilin. Furthermore, an increase in the expression of the phosphorylated form of LMWPTP, which was followed by an augment in its catalytic activity, was observed. Of particular importance, these responses occurred in an intracellular milieu characterized by elevated levels of reduced glutathione (GSH) and increased expression of the antioxidant enzymes glutathione peroxidase and glutathione reductase. Altogether, our results suggest that hyperosmostic stress provides a favorable cellular environment to the activation of LMWPTP, which is associated with increased expression of antioxidant enzymes, high levels of GSH and inhibition of Src kinase. Finally, the real contribution of LMWPTP in the hyperosmotic stress response of keratinocytes was demonstrated through analysis of the effects of ACP1 gene knockdown in stressed and non-stressed cells. LMWPTP knockdown attenuates the effects of sorbitol induced-stress in HaCaT cells, mainly in the status of Src kinase, Rac and STAT5 phosphorylation and activity. These results describe for the first time the participation of LMWPTP in the dynamics of cytoskeleton rearrangement during exposure of human keratinocytes to hyperosmotic shock, which may contribute to cell death.

## Introduction

Human epidermis is a multilayered epithelium that forms the interface between the environment and the organism. Due to its unique mechanical and biochemical properties, the epidermis provides the barrier of body’s defense against environmental and physiological stressful conditions, such as UV exposition, dehydration, heat or other damaging insults such as inflammation [1]. Keratinocytes, the major constituent of this tissue, exhibit a controlled program of differentiation that allows the dynamic and efficient recovery of skin layers [2]. However, changes in epidermal homeostasis may occur even in response to metabolically inert compounds, such as sorbitol, which is known to cause hyperosmotic stress effects in the skin [3,4]. In this condition, epidermal keratinocytes can trigger diverse intracellular signaling cascades involved in the control of proliferation and differentiation [5,6]. Moreover, depending on the intensity and duration of the stimulus, keratinocytes may undergo programmed cell death [7].

Hyperosmotic stress initiates several adaptive responses, among them, the remodeling of the cytoskeleton is of particular importance due to its role in gene transcription and signal transduction [8,9]. In this context, keratinocytes are able to respond to hyperosmolarity through cytoskeleton remodeling [10], even though the underlying mechanisms and signaling network associated with this event have not been sufficiently defined. Recently, it was demonstrated that hyperosmotic stress induces a rapid, sustained and reversible phosphorylation of the actin-regulatory protein cofilin in kidney epithelial cells, and that this effect was mediated by the small GTPase Rho [11]. Also, mitochondrial translocation and oxidation of cofilin were shown to play a role in oxidant-induced apoptosis through loss of mitochondrial integrity [12,13]. Clearly, different biochemical mechanisms mediate cytoskeleton alterations during apoptosis induction, such as oxidation and phosphorylation of cytoskeletal proteins.

Reversible phosphorylation of tyrosine residues in proteins plays a key regulatory function in cell physiology. Protein tyrosine phosphatases (PTP) act together with protein tyrosine kinases (PTK) to regulate protein phosphotyrosine levels in signaling molecules, thereby mediating specific changes in cellular responses, such as cell growth and differentiation, cell cycle, metabolism, and cytoskeletal function [14]. In particular, the 18 kDa enzymes of the low molecular weight PTP (LMWPTP) family, which is encoded by the acid phosphatase locus 1 gene (*ACP1*), have been considered to play key regulatory functions in signaling pathways involved in cytoskeleton rearrangement [15,16]. Modulation of its activity is based on phosphorylation/dephosphorylation of tyrosine residues and reversible oxidation of catalytic cysteine residues. Whereas phosphorylation of the conserved adjacent tyrosines, Tyr131 and Tyr132, modulates LMWPTP activity and substrates selectivity to different extents, the generation of intracellular reactive oxygen species (ROS) induces the inhibition of the enzyme through direct and indirect effects. Indeed, ROS promotes the reversible oxidation of key cysteine residues of the catalytic site leading to the inactivation of the enzyme. This event is also reinforced by the actions of ROS upon PTK since it is known that oxidation of amino acid residues in PTK promotes the activation of such enzymes [15,16,17,18]. In this respect, it has been previously demonstrated that LMWPTP oxidation leads to its inactivation and potentiates the action of PTK such as Src that can phosphorylate the phosphatase in Tyr132, contributing to its inactivation [19,20,21,22].

Herein, our aim was to investigate some events involved in keratinocytes response to hyperosmotic stress induced by an osmotically nonionic compound, sorbitol, using a human keratinocyte cell line, HaCaT. In particular, given the activities displayed by LMWPTP in regulating several cellular events, including cytoskeleton functions, its involvement in such responses was studied.

## Materials and Methods

### Materials

Sorbitol, bovine serum albumin (BSA), [3-(4,5-dimethylthiazol-2-yl)-2,5-diphenyltetrazolium bromide] (MTT), trypan blue, neutral red and Protein Tyrosine Phosphatase Assay Kit—PTP-101 were purchased from Sigma Chemical Co. (St. Louis, MO, USA). Antibodies against β-actin, catalase, glutathione peroxidase (GPX), glutathione reductase (GR), phospho-PP2A (Ser8), PARP-1 [poly(ADP-ribose) polymerase], phalloidin conjugated with Alexa Fluor 488 were purchased from Santa Cruz Biotechnology (Santa Cruz, CA, USA). Anti-cleaved caspase-3 (Asp175) antibody conjugated with Alexa Fluor 488, anti-glyceraldehyde 3-phoshate dehydrogenase (GAPDH), anti-cofilin, anti-STAT5, anti-phospho-STAT5 (Tyr694), anti-phospho-cofilin (Ser3), anti-total Src, anti-phospho-Src (Tyr416), anti-phospho-Src (Tyr527), anti-Rac-1, anti-RhoA, anti-PKAα, and horseradish peroxidase (HRP)-linked secondary anti-rabbit and anti-mouse antibodies were bought from Cell Signaling Technology (Beverly, MA, USA). Alexa Fluor 594-conjugated wheat germ agglutinin (WGA594), Protein A-Sepharose, 4′,6-diamidino-2-phenylindole (DAPI) and Alexa Fluor 594 goat anti-rabbit IgG antibody were purchased from Life Technologies/Molecular Probes, Inc. (Eugene, OR, USA). Anti-LMWPTP and HRP-linked secondary anti-goat antibodies were obtained from Abcam (San Francisco, CA, USA). Paraformaldehyde and Fluoromount G were from Electron Microscopy Sciences (Hatfield, PA, USA), and annexin V conjugated to allophycocyanin (APC) was from BD Biosciences (San Jose, CA, USA). Fugene HD Transfection Reagent was obtained from Promega (Madison, WI, USA). All the other chemicals and reagents used in this study were of analytical grade, purchased from commercial sources.

### Methods

#### Cell culture and sorbitol-induced hyperosmotic stress

The human keratinocyte cell line HaCaT [23,24] was gently provided by Dr. Liudmila Kodach (Amsterdam Medical Center, Amsterdam, The Netherlands). These cells were cultured in Dulbecco’s Modified Eagle’s medium (DMEM; Sigma Chemical Co.) supplemented with 10% fetal calf serum (FCS; Gibco, Grand Island, NY, USA), 100 U/ml of penicillin and 100 μg/ml of streptomycin at 37°C in a humidified atmosphere containing 5% CO_2_. Viability and cell density were determined by the trypan blue dye exclusion test.

Hyperosmotic stress was induced in keratinocytes (3.5 x 10^4^ cells/ml) seeded in sextuplicates into 96-well plates in DMEM supplemented with 10% FCS. After 48 h of incubation at 37°C in a humidified atmosphere containing 5% CO_2_, the medium was replaced with DMEM containing BSA (0.5 mg/ml) and cells were incubated for another period of 24 h. Cells were then treated with different concentrations of sorbitol (0.2–2 M), diluted in serum-free culture medium, while control samples were treated with the corresponding volume of serum-free culture medium. All samples were incubated for 2 h at 37°C in a humidified atmosphere containing 5% CO_2_. After treatment, cells were harvested for analysis.

Effects of sorbitol on cell viability were evaluated by the MTT reduction assay (MTT), the neutral red uptake (NRU) and the total nucleic acid content (NAC), as previously described [25,26,27]. The results were expressed as percent of the cell viability of untreated control cells (100%).

#### Knocking down of *ACP1* gene and immunofluorescence

To knockdown *ACP1* gene, two different siRNAs (siAPC1-CIII and siACP1-CV) were designed based on target sequences described previously in [28] and synthesized forming siRNA duplexes (Sigma-Aldrich Co., St. Louis, MO, USA). MISSION siRNA Universal Negative Control #1 (Sigma-Aldrich Co.) was used as a control (Scramble). Each target sequence does not show significant homology to other human gene sequences. HaCaT cells were seeded at 80% confluence and transfected using Fugene HD Transfection Reagent, with 40 nM of each siRNA duplex, separately, according to the manufacturer (Promega). Cells were incubated for 5 h before hyperosmotic stress was carried out. To verify the effectiveness of RNA interference, total protein was extracted and western blotting was performed.

For immunofluorescence, cells grown on glass coverslips were fixed with 2% paraformaldehyde for 30 min, washed with 0.1 mol/l glycine, and permeabilized with 0.01% saponin. After 30 min, cells were washed with PBS, pH 7.4, and incubated with Alexa Fluor 488-conjugated phalloidin in PBS/1% BSA for 2 h. Cells were then stained with primary anti-Rac-1 antibody for 2 h, washed and incubated with Alexa Fluor 594 goat anti-rabbit IgG antibody for 2 h. After washing, the nuclei were stained with DAPI for 30 min. Cells were washed and coverslips were mounted on glass slides using Fluoromount-G and examined in a laser scanning confocal microscope (Leica SP8, Germany). Alexa Fluor 488 was excited at 493 nm and emission was captured at 520 nm. Alexa Fluor 594 was excited at 590 nm and the emission was detected at 619 nm. For DAPI, the excitation wavelength was 405 nm and the emission was captured at 461 nm. Images presented in the figures are representative of at least three experiments.

#### Glutathione assay

Keratinocytes (5 x 10^6^ cells) were treated with 1 M sorbitol for 2 h, followed by cell washing with physiological solution and lysis with 2 ml of water. A volume of 3 ml of precipitant solution (1.67 g of glacial metaphosphoric acid, 0.2 g of EDTA and 30 g of NaCl in 100 ml of deionized water) was added to the lysate. After 5 min, the mixture was centrifuged and the GSH concentration was determined according to Torsoni et al. [29] with some modifications. Briefly, the reaction of 1.1 ml of the supernatant with 5,5’-dithio-bis-2-nitrobenzoic acid (DTNB) was done in the presence of 0.2 M phosphate buffered saline (PBS), pH 8.0. After 5 min of reaction, absorbance was measured at 412 nm and the GSH content calculated in relation to the control (ε = 13.6 mol^-1^ cm^-1^). Control cells were treated with medium only. Viability was determined by the trypan blue exclusion test.

#### Assessment of redox status

HaCaT cells were treated with 1 M sorbitol in DMEM medium for 2h. After treatment, the cells were rinsed three times with cold PBS, ressuspended in 400 μl of ice cold lysis buffer [10 mmol/l phosphate buffer (pH 7.0)] and sonicated in an ice bath for 15 s. Thereafter, the total amount of protein extracts were cleared by centrifugation and the supernatant was collected and submitted to protein quantification [30] followed by assessment of the redox status using different assays. Catalase activity was measured by monitoring H_2_O_2_ decomposition determined by changes in A_230_ nm during 30 min incubation in reaction medium containing 50 mmol/l sodium phosphate buffer (pH 7.0), 10 mmol/l H_2_O_2_ and enzyme sample [31]. Glutathione S-transferase (GST) activity was assayed based on the conjugation of CDNB with glutathione and changes were monitored at A_340_ nm [32]. Oxidative stress was assessed based on the quantification of malondialdehyde (MDA), a secondary product of lipid peroxidation. The lipid peroxidation product MDA was determined by incubation with MPO (n-methyl-2-phenylindole; Sigma), followed by measurement of the absorbance at 590 nm [33]. The results were expressed as nmol. μg prot.^-1^.min^-1^ for catalase and GST and nM. μg prot.^-1^ for MDA. All the antioxidant enzyme activities were expressed as ΔOD⁄min⁄μg of protein and the absorbances were measured with an enzyme linked immunosorbent assay (ELISA) plate reader.

#### Evaluation of phosphatidylserine exposure by confocal microscopy

HaCaT cells were seeded onto glass coverslips and incubated for 72 h at 37°C in a humidified atmosphere containing 5% CO_2_. Afterwards, the plasma membranes were stained with 10 μg/ml Alexa Fluor 594-conjugated WGA (WGA594) for 30 min. After washing in culture medium, HaCaT cells were treated with 1 M sorbitol in DMEM medium containing 5 μl annexin V-APC during 2 h. Cells undergoing phosphatidylserine exposure were then microscopically examined using an inverted laser scanning confocal microscope LSM 510 (Zeiss, Germany). The WGA594 was excited with a HeNe laser (excitation = 546 nm) and light emission was detected at 560–610 nm. The annexin V-APC was excited with a HeNe laser (excitation = 633 nm) and light emission was detected at 635–700 nm. Temporal images were captured with intervals of 10 min. The intensity of annexin V-APC was quantified using Examiner 4.2 (Zeiss) and was normalized using WGA594 intensity. Images were prepared using the programs Examiner 4.2 (Zeiss) and Adobe Photoshop 9.2.

#### Analysis of caspase-3 activity by flow cytometry

The endogenous levels of the large fragment (17/19 kDa) of activated caspase-3 were evaluated by flow cytometry. After treatment with 1 M sorbitol for 2 h, HaCaT cells were harvested by centrifugation, washed with ice-cold PBS and fixed in paraformaldehyde 2% in PBS (v/v) for 30 min. Cells were then permeabilized in PBS containing 0.01% saponin for 15 min and blocked in PBS containing 1% BSA for 30 min at room temperature. Afterwards, 10 μl of cleaved caspase-3 (Asp175) Alexa Fluor 488-conjugated antibody were added and cells were incubated in the dark at room temperature for 1 h. The antibodies were diluted according the manufacturer’s instructions (Cell Signaling Technology). Cells were then washed in PBS containing 1% BSA, resuspended in 300 μl PBS and analyzed (10.000 events were collected per sample) in a FACSCalibur flow cytometer (Becton Dickinson, CA, USA) using the CellQuest software (BD).

#### Immunoblotting

After hyperosmotic stress, HaCaT cells were washed in ice-cold PBS and protein extracts were obtained using a lysis buffer [50 mM Tris-HCl, pH 7.4, 1% Tween 20, 0.25% sodium deoxycholate, 150 mM NaCl, 1 mM EGTA, 1 mmol/l Na_3_VO_4_, 1 mM NaF and protease inhibitors (1 μg/ml aprotinin, 10 μg/ml leupeptin, and 1 mM 4-(2-aminoethyl) benzenesulfonyl fluoride)] for 2 h on ice. Protein extracts were cleared by centrifugation and protein concentrations were determined using the Lowry protein assay [30]. An equal volume of 2X SDS gel loading buffer (100 mM Tris-HCl, pH 6.8, 200 mM dithiothreitol, 4% SDS, 0.1% bromophenol blue and 20% glycerol) was added to the samples and boiled for 5 min. Equal amounts of protein (50 μg) were loaded onto SDS-PAGE and blotted onto PVDF membranes (Millipore, Bedford, MA, USA). Membranes were blocked in 1% fat-free dried milk or 2% BSA in Tris-buffered saline (TBS) with 0.05% Tween 20 (TBST) and incubated overnight at 4°C with appropriate primary antibody at 1:1000 dilution. After washing in TBST, membranes were incubated with appropriate HRP-linked secondary antibodies, at 1:5000 dilutions, in blocking buffer for 1 h. Immunoreactive bands were detected with enhanced chemiluminescence kit.

#### LMWPTP immunoprecipitation

After hyperosmotic stress, cells were lysed in ice-cold lysis buffer [50 mM HEPES (pH 7.5), 150 mM NaCl, 1.5 mM MgCl_2_, 1 mM EGTA, 10% glicerol, 1% Triton X-100, 1 mM PMSF, 1 μg/ml leupeptin and 1 μg/ml aprotinin] for 2 h in ice. Protein extracts were cleared by centrifugation and protein concentrations were determined using the Lowry protein assay [30]. Lysates were then incubated (rotatory mixing) for 1 h with specific antibody against LMWPTP and Protein A-Sepharose at 4°C. After centrifugation, immunoprecipitates were washed three times with HNTG sample [20 mM HEPES (pH 7.5), 150 mM NaCl, 10% glicerol and 0.1% Triton X-100] and used for enzymatic assay, SDS-PAGE and immunoblotting as described above.

#### LMWPTP activity assay

For determination of the tyrosine phosphatase activity, the immunoprecipitates were washed and resuspended in 0.1 mol/l sodium acetate buffer, pH 5.5. The LMWPTP activity was analyzed using the Protein Tyrosine Phosphatase Assay Kit—PTP-101, according to the manufacturer’s instructions (Sigma Chemical Co.). Enzyme activities were calculated in pmol PO_4_^2-^ mg^-1^ min^-1^, and expressed relative to control.

#### Statistical analysis

All experiments were performed at least three times. Results were expressed as mean ± standard deviation. Statistical analysis was performed by the Student’s *t* test, or analysis of variance (ANOVA) followed by the *post hoc* Tukey test when more than two groups were compared, using Instat software (SigmaStat for Windows version 3.1, Systat Software Inc., USA). Differences were considered significant at P < 0.05, representing two-sided test of statistical significance. Densitometric analysis of blots was performed using the Scion Image software.

## Results

### Hyperosmotic stress induces keratinocyte death

**Fig 1:**
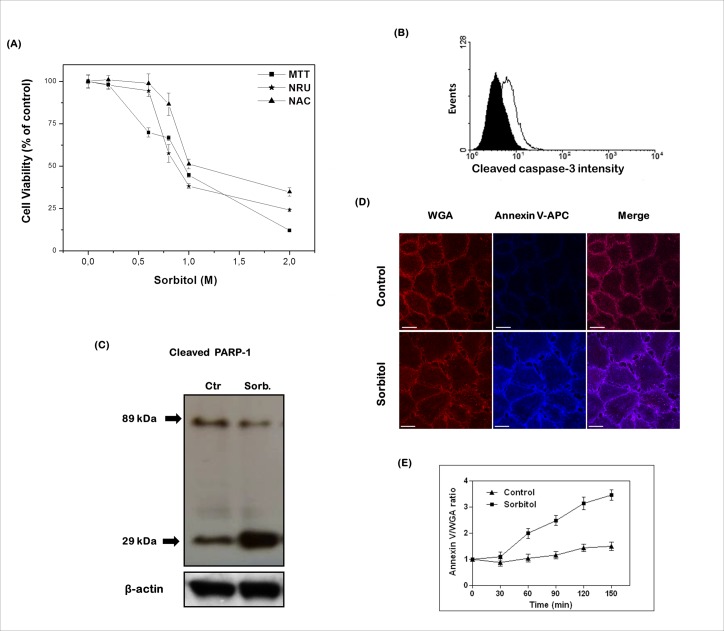
Sorbitol cytotoxicity in HaCaT cells involves induction of apoptosis. **(A)** Confluent layers of HaCaT cells were challenged with different concentrations of sorbitol (0.2–2 M in serum-free culture medium) for 2 h, and cellular viability was assessed by three different endpoints assays [MTT reduction (MTT), neutral red uptake (NRU) and nucleic acid content (NAC)]. The results were expressed as percentage of control cell viability (100%) and represented as mean ± SD of three independent experiments run in quadruplicate. HaCaT cells exposed to 1 M sorbitol for 2 h exhibited typical signs of apoptosis. **(B)** Increased fluorescence intensity of the large fragment (17/19 kDa) of activated caspase-3 detected by flow cytometry in stressed (open histogram) relative to control (black histogram) cells. One representative histogram of three independent experiments for each sample is presented. **(C)** Increased levels of the small fragment of cleaved PARP-1 by immunoblotting. Equal amounts of total protein (50 μg) from cell lysates were loaded per lane and blotted with specific antibodies. One representative immunoblot of three independent experiments is presented. **(D)** Increased phosphatidylserine exposition on the extracellular face of the membranes of stressed compared to control cells, as shown by confocal microscopy analysis of membrane labeled with WGA594 (red) and phosphatidylserine exposure labeled with annexin V-APC (blue). Images are representative of three independent experiments at the end of treatment (2 h). Bars = 20 μm. **(E)** Time-dependent increase in the relative fluorescence intensity of annexin V/WGA was quantified from temporal images captured with intervals of 10 min in experiment depicted in **(D)**, using the Examiner 4.2 software. The results were expressed as mean ± standard deviation of the relative fluorescence intensities of each cell per field of view. At least three different fields were analyzed in three independent experiments.

It has been previously demonstrated the pro-apoptotic effects of hyperosmotic stress on human immortalized HaCaT keratinocytes, a well studied model of proliferating non-tumorigenic epidermal cells [34]. Herein, HaCaT cells were exposed for 2 h to a nonionic osmolyte, sorbitol, to induce hyperosmotic stress. Multiple, mechanistically different parameters were used to assess HaCaT cell viability: the total nucleic acid content (NAC), neutral red uptake (NRU) and reduction of the MTT [25,26,27]. As shown in Fig. 1A, the viability of HaCaT cells decreased in a dose-dependent manner in response to increasing concentrations of sorbitol (0.2–2 M). According to MTT and NRU assays, the 50% reduction in cell viability (IC_50_ value) was obtained with a concentration of sorbitol around 1 M. Importantly, a similar cytotoxicity profile was observed by predicting the cell number, using the NAC assay, as seen for the MTT and NRU approaches, indicating that sorbitol induces cell death. Next, we evaluated the characteristics of sorbitol-induced HaCaT cell death by examining typical biochemical and morphological alterations associated with apoptosis. Our results demonstrated that hyperosmotic stress promoted the cleavage and activation of caspase-3, as demonstrated by the increased fluorescence intensity of its large fragment (17/19 kDa) detected directly by flow cytometry (Fig. 1B). Moreover, this is in agreement with the increased cleavage of the caspase-3 substrate, PARP-1, as indicated by the higher levels of its small fragment in immunoblot analysis (Fig. 1C).

Since translocation of phosphatidylserine from the inside to the outside surface of the plasma membrane is an important indicator of apoptosis induction, we followed phosphatidylserine exposition on the extracellular face of the membrane by annexin V staining. Confocal microscopy revealed that hyperosmolarity caused a marked increase in peripheral labeling that is consistent with an enhanced annexin V staining in a narrow line along the cell membrane (Fig. 1D). Furthermore, a time-dependent increase in annexin V/WGA ratio was found, indicating a 2-fold increase in annexin V fluorescence intensity relative to control after 1 h of stress exposure as long as the maximum increase (3-fold increase) was observed at 2 h (Fig. 1E). Together, these results corroborate with those previously reported by Diker-Cohen et al. [2], indicating that hyperosmolarity-provoked HaCaT cell death is mediated by apoptosis.

### Modulation of expression/activation of signaling proteins associated with cytoskeletal modeling

**Fig 2:**
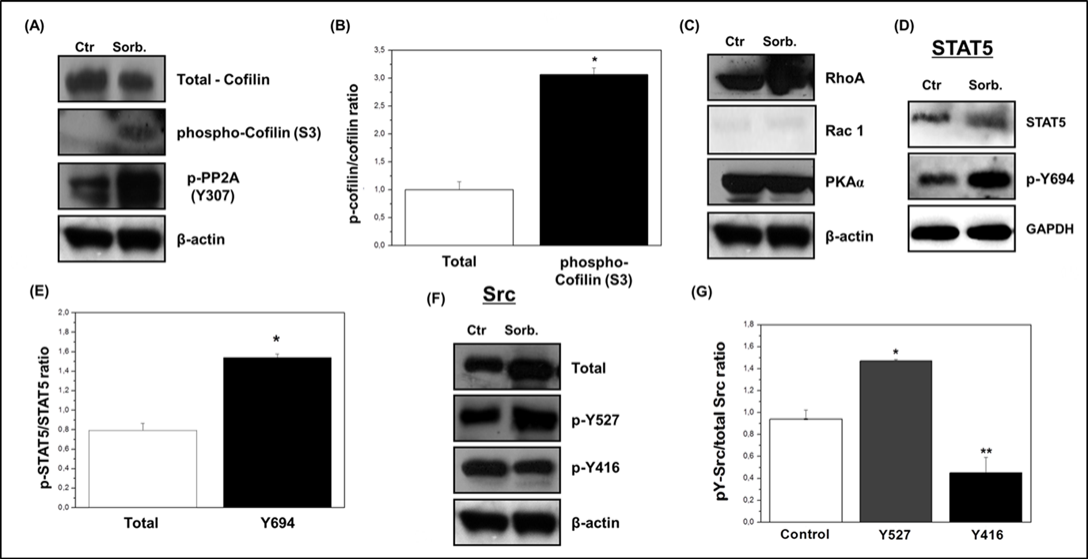
Effects of hyperosmotic stress on regulatory enzymes associated with cytoskeletal remodeling. HaCaT cells were exposed to 1 M sorbitol for 2 h, harvested and lysed as described in Materials and Methods. Equal amounts of total protein (50 μg) from cell lysates were loaded per lane and blotted with specific antibodies. One representative immunoblot of three independent experiments is presented. β-actin or GAPDH was used as loading control. **(A)** Hyperosmotic stress induces cofilin phosphorylation and PP2A inhibition. To check for total cofilin expression and equality of protein loading, antibodies against cofilin and β-actin were used. **(B)** Densitometric analysis of the hyperosmolarity-induced cofilin phosphorylation. Data are expressed as phospho-cofilin/cofilin ratio normalized to the protein ratio of controls (1). **(C)** Hyperosmotic stress causes significant increases in the expression of RhoA and Rac-1, while levels of PKAα remained unchanged. **(D)** Hyperosmotic stress is associated with STAT5 activation. **(E)** Densitometric analysis of immunoblots was expressed as the relative intensity of phospho-YSTAT5/STAT5 ratios normalized to the protein ratio of controls (1). **(F)** Sorbitol promotes inactivation of Src kinase. Despite the increase in protein levels of total Src kinase, a significant decrease in the phosphorylation of Y416 located on the activation loop of the kinase, and an increase of Y527, which corresponds to its inhibitory site, occur. (E) Densitometric analysis of immunoblots was expressed as the relative intensity of phospho-YSrc/total Src ratios normalized to the protein ratio of controls (1). Results were represented as mean ± standard deviation of three independent experiments. *P < 0.05 and **P < 0.001 compared with control.

Cofilin has been implicated as an important regulator of cell fate in response to stress, due to its role in modulating actin-stressed filaments. The effects of osmotic stress on cofilin status were investigated in keratinocytes exposed to the IC_50_ concentration of sorbitol. As can be seen in Fig. 2A, the hyperosmotic challenge caused a substantial elevation of cofilin phosphorylation at Ser3 that was accompanied by a slight decrease in its expression, leading to a 3-fold increase in the phospho-cofilin/cofilin ratio relative to control (Fig. 2B). Concomitantly, a significant rise in the phosphorylation level of the inhibitory site of PP2A (Tyr307) was observed in this condition (Fig. 2A). These results are consistent with the view that the inhibition of PP2A may guarantee high levels of phosphorylated cofilin in treated cells since this protein is a recognized substrate of PP2A [35].

It has been shown that hyperosmotic stress induces cofilin phosphorylation through activation of the Rho/ROCK/LIMK pathway. This event has been considered as a key contributor for the increase in F-actin during the stress periods. Indeed, data from the literature have shown that hyperosmotic stress induces LIMK phosphorylation leading to a rapid and sustained Rho activation. In agreement, Rho or ROCK inhibition can avoid the shrinkage-induced cofilin phosphorylation and LIMK activation [36,11]. In an opposite way, activation of Rac signaling pathways inhibits the activity of the small GTPase-RhoA through activation of Rho-GAP activity [37,38]. In this way, the balance of Rac and Rho activities is fundamental and decisive for cytoskeleton rearrangement, alterations in cellular morphology and migratory behavior mainly in the determination of cofilin phosphorylation status induced by hyperosmotic stress periods [11,39]. Thus, in addition to cofilin, we analyzed by immunoblotting the expression/activation status of the small GTPases RhoA, Rac-1 and, also, PKAα. Our data showed that RhoA expression was significantly increased, while levels of Rac-1 and PKAα remained unchanged (Fig. 2C) in our experimental model. The activity of Rho-A is influenced by the activity and functional status of Rho-GAP. In its phosphorylated form Rho-GAP is active, while its dephosphorylation inactivates the protein that no more can hydrolyze de GTP in Rho, thus maintaining its active state. As demonstrated later in our work, the tyrosine phosphatase LMWPTP is activated by the hyperosmotic stress induced by sorbitol in HaCaT cells and this enzyme is able to dephosphorylate Rho-GAP and activate RhoA, suggesting that besides the increase in its expression, Rho-A possibly presents an increase in its activity associated with the activity of LMWPTP. Finally, our results reinforce the role of RhoA-induced intracellular signaling in the induction of cofilin phosphorylation and associate these events with the cytoskeletal rearrangements induced by the hyperosmotic stress.

### Hyperosmotic stress is associated with STAT5 activation

It is well described that when mammalian cells are subjected to osmotic or oxidative stress they respond to such stresses through activation of JAK/STAT signaling pathways and maintenance of its activity through inhibition of protein tyrosine phosphatases such as PTP3. As recently demonstrated, in *Dictyostelium* cells the Ser448 and Ser747 phosphorylation of the protein-tyrosine phosphatase-3 (PTP3) is induced by a variety of stresses resulting in inhibition of its phosphatase activity and, consequently, maintenance of its substrate, STATc, activity [40]. In agreement with the data from literature, the results showed that after hyperosmotic stress both expression and activity (phosphorylation status at Tyr694) of STAT5 were increased (Fig. 2D and E).

### Hyperosmotic stress induces inhibition of Src activity

It is well known that Src is a pivotal mediator of cell adhesion and cytoskeleton rearrangements, affecting downstream protein activation, such as Rho and ROCK family members [41,42]. Thus, we next checked the expression and phosphorylation status of Src. Although sorbitol treatment promoted an increase in protein levels, a significant reduction (>50%) in the phosphorylation of Tyr416 on Src was observed. In addition, an increase at Tyr527 phosphorylation, which corresponds to the inhibitory site of the kinase, also occurred (Fig. 2F and G). The balance of these phosphorylations alters Src conformation leading to its inactivation, which may contribute to cell death. Therefore, a specific regulatory mechanism of Src kinase activation, acting on Tyr527 and Tyr416 located in the activation loop of the enzyme may exist in the response of keratinocytes to hyperosmotic stress conditions.

### Hyperosmotic stress induces LMWPTP activation in keratinocytes

**Fig 3:**
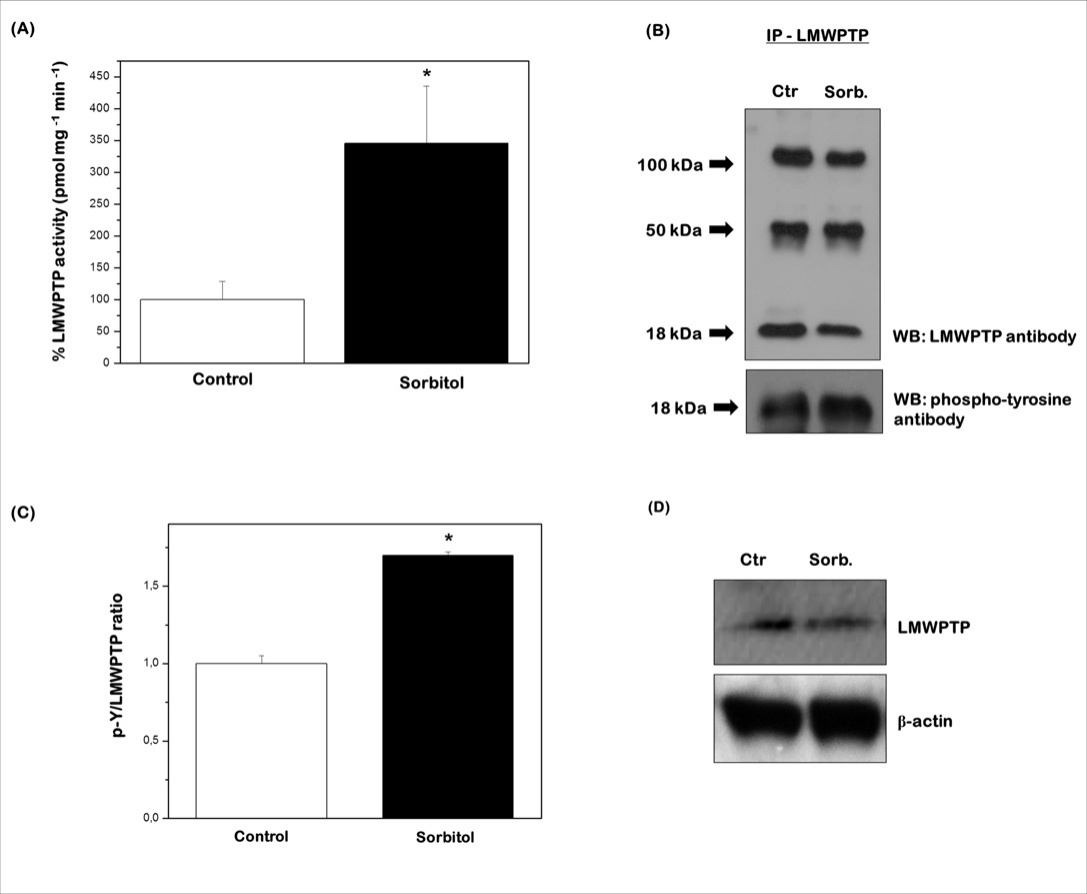
LMWPTP is markedly activated in stressed keratinocytes. HaCaT cells were exposed to 1 M sorbitol for 2 h, harvested and lysed. **(A)** Hyperosmotic stress increases the activity of immunoprecipitated LMWPTP (IP-LMWPTP). *P < 0.001 compared with control. **(B)** LMWPTP was immunoprecipitated and an anti-phosphotyrosine immunoblotting was performed. The blot was then stripped and reprobed with anti-LMWPTP antibody for normalization by densitometric analysis. **(C)** Hyperosmolarity increases the relative phosphorylated/non-phosphorylated LMWPTP ratios normalized to the protein ratio of controls (1). Results were represented as mean ± standard deviation of three independent experiments. *P < 0.001 compared with control. **(D)** Sorbitol does not affect LMWPTP expression. Equal amounts of total protein (50 μg) from cell lysates were loaded per lane and blotted with anti-LMWPTP antibody. β-actin was used as loading control. One representative immunoblot of three independent experiments is presented.

Given the general importance of PTP activity in the regulation of many crucial events associated with cellular physiology and, in particular, the ability of the LMWPTP to interact with Src kinase [18,20,43], the possible involvement of this phosphatase in the control of keratinocyte response to hyperosmotic stress was evaluated. To address this issue, we first compared LMWPTP expression and activity between control and stressed keratinocytes. HaCaT cells were treated for 2 h with 1 M sorbitol or medium and the activity of immunoprecipitated-LMWPTP (IP-LMWPTP) was assessed. The results showed that hyperosmotic stress caused a large increase (3.5-fold) in LMWPTP specific activity (Fig. 3A) compared with the control group. Furthermore, immunoblot analysis of Tyr phosphorylation status on IP-LMWPTP also corroborates this result, as the level of phospho-Tyr in IP-LMWPTP was increased in stressed keratinocytes (Fig. 3B-C). In addition, the expression levels of LMWPTP were evaluated in total protein extracts by immunoblotting and no changes in the amounts of LMWPTP were induced by sorbitol exposition (Fig. 3D). Altogether, these results indicated a positive action of hyperosmotic stress in the modulation of LMWPTP activity.

### Hyperosmotic stress modulates antioxidant defenses in keratinocytes

A role for oxidative signaling in hyperosmotic stress has been suggested in different cell models [44]. Importantly, beside its phosphorylation status, the redox environment also modulates LMWPTP activity by oxidizing a cysteine residue in the active site that is crucial for its catalytic activity. Thus, in view of the importance of the cellular redox status in modulating the activity of PTP in general [14,15], including the LMWPTP [27], analyses of the expression and activity of antioxidant enzymes in sorbitol-treated HaCaT cells were performed by western blotting and activity assays.

**Fig 4:**
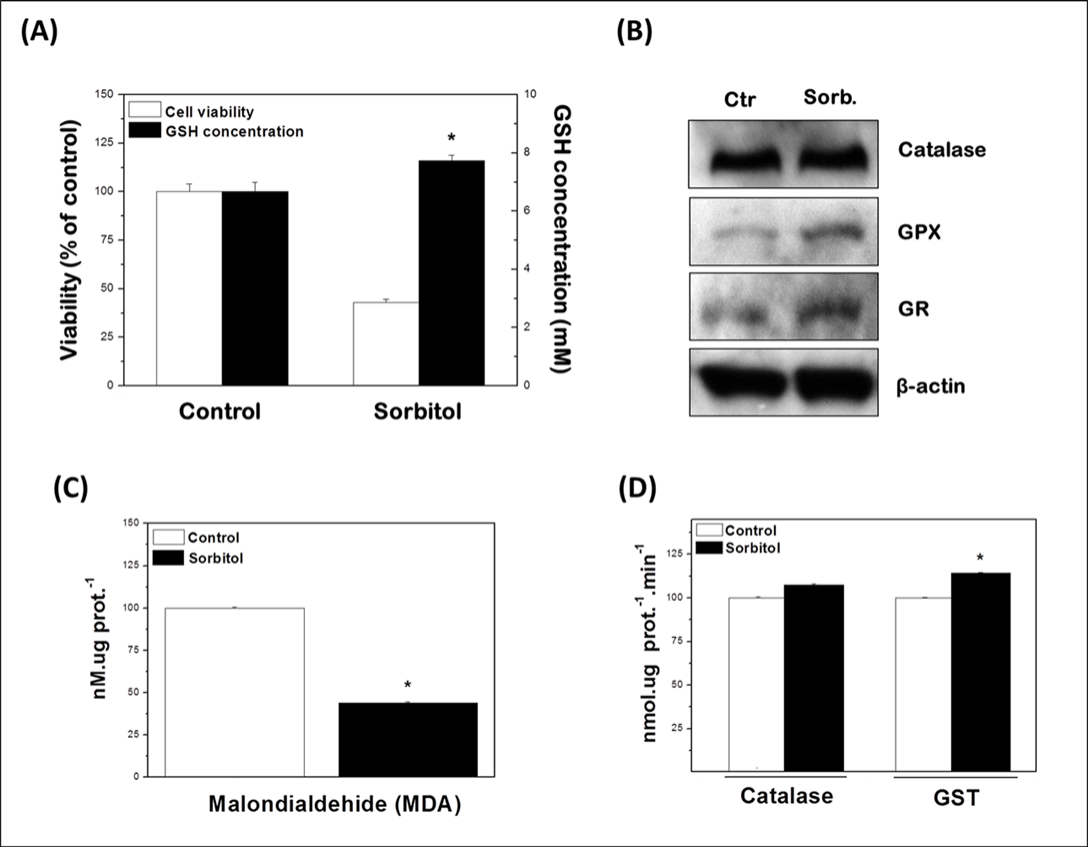
Changes in the cellular redox status induced by hyperosmotic stress. **(A)** Hyperosmotic stress dramatically increases GSH concentration in HaCaT cells, especially considering the reduced number of viable cells after stress. Viability was determined by the trypan blue exclusion method. All the results were expressed as percentage of control (100%) and represented as mean ± SD of three independent experiments run in triplicate. *P < 0.05 compared with control. **(B)** Hyperosmotic stress causes significant increases in the expression of glutathione peroxidase (GPX) and glutathione reductase (GR), while levels of catalase remained unchanged. After hyperosmotic stress, cells were harvested and lysed. Equal amounts of total protein (50 μg) from cell lysates were loaded per lane and blotted with specific antibodies. One representative immunoblot of three independent experiments is presented. β-actin was used as loading control. **(C)** The influence of hyperomostic stress on the malondialdehyde content (nM.ug prot.^-1^) and **(D)** glutathione-S-transferase and catalase activity (nmol.ug prot.^-1^.min^-1^). Results were represented as mean ± standard deviation of three independent experiments. *P < 0.05 compared with control.

As indicated in Fig. 4 (Fig. 4A, 4B) treatment of cells with sorbitol had no difference in catalase expression but increased the levels of reduced glutathione (GSH) and upregulated the expression of glutathione reductase (GR) and glutathione peroxidase (GPX). Also, although no significant differences in catalase activity were observed after exposition of cells to sorbitol, glutathione S-transferase (GST) has its activity increased in such condition (Fig. 4D). GST is a member of the superfamily of multifunctional dimeric proteins involved in the cellular detoxification of cytotoxic and genotoxic compounds and in the prevention of tissue oxidative damages. In addition to their role in catalyzing the conjugation of electrophilic substrates to GSH, these enzymes also carry out a range of other functions in cells, such as the removal of reactive oxygen species and regeneration of S-thiolated proteins (both of which are consequences of oxidative stress), catalysis of conjugations with endogenous ligands, and catalysis of reactions in metabolic pathways not associated with detoxification [45,46]. Finally, the effectiveness of glutathione-based antioxidant defense mechanism is reinforced by the diminished levels of lipid peroxidation in treated cells, as indicated by the reduced concentration of MDA, a secondary product of lipid peroxidation (Fig. 4C).

Previous work has pointed out the importance of the glutathione/glutathione reductase/NADPH system in the reversible oxidation of LMWPTP, underlying GSH as the major non-protein thiol in mammalian cells with protective action against the irreversible oxidation of this phosphatase [47]. Based on the results shown here, it is possible to conclude that antioxidant defense mechanisms guarantee the availability of GSH to maintain LMWPTP cysteine residue in its reduced form, thus contributing for the activity of this enzyme.

### Role of LMWPTP in hyperosmotic stress

**Fig 5:**
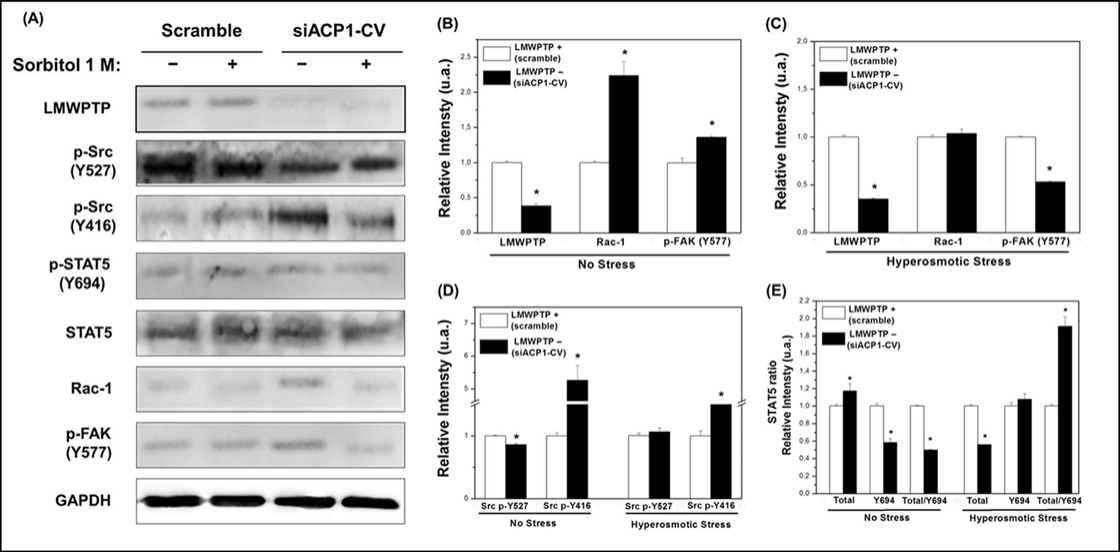
Effect of LMWPTP knockdown with siACP1-CV on expression/activity of Rac-1, Src, FAK and STAT5. HaCaT cells were transfected with siACP1-CV 40 nM, exposed to 1 M sorbitol for 2 h, harvested and lysed as described in Materials and Methods. Equal amounts of total protein (50 μg) from cell lysates were loaded per lane and blotted with specific antibodies. One representative immunoblot of three independent experiments is presented. GAPDH was used as loading control. The effects of *ACP1* knockdown in Rac-1, Src, FAK and STAT5 proteins were evaluated in HaCaT cells before and after exposition to sorbitol-induced stress **(A)**. **(B-E)** Densitometric analysis of the results normalized to the protein content of LMWPTP-expressing cells (value equal to 1) comparing stressed and non-stressed cells. Results were represented as mean ± standard deviation of three independent experiments. *P < 0.001 compared with control.

As demonstrated by the results obtained in this work, it is clear the effects of hyperosmotic stress in keratinocytes LMWPTP activity. However, while the data shows the stimulatory action of hyperosmotic stress in the activity of LMWPTP, nothing is known about the effects of this activation in the molecular events associated with HaCaT response to such stress. To address this point, the expression of this phosphatase was knocking down in HaCaT cells using specific small interfering RNA against ACP1 gene, and its effects were analyzed before and after cell treatment with 1 M sorbitol. Efficiency of knockdowns was confirmed by the reduced levels of LMWPTP protein expression, obtaining better results with transfection of cells with 40 nM of siACP1-CV (Fig. 5A and data not shown). Thus, all experiments involving siRNA knockdown were conducted using 40 nM siACP1-CV.

The analysis of data obtained in non-stressed HaCaT cells showed that this phosphatase is, indeed, an active modulator of Src kinase, FAK and STAT5 expression/activity. Comparing the phosphorylation status of residues Tyr416 and Tyr527 before and after LMWPTP knockdown, it is clear that this phosphatase contributes for inactivation of Src kinase in normal, non-stressed cells. In fact, after LMWPTP knockdown, the cells presented a significant increase in Tyr416 phosphorylation, while a reduction in the phosphorylation levels at Tyr527 occurred (Fig. 5A and D). In agreement, LMWPTP knockdown resulted in an increase in Tyr577 phosphorylation of FAK protein (Fig. 5B), a known site of Src kinase activity. These results demonstrated that LMWPTP is an important regulator of Src activity in HaCaT cells, thus corroborating with other works showing Src kinase as a substrate of this phosphatase [43]. Interestingly, LMWPTP knockdown attenuates the effects of sorbitol induced-stress in HaCaT cells (Fig. 5A), mainly in the status of Src kinase phosphorylation and activity. As demonstrated previously, treatment of HaCaT cells with sorbitol induces a reduction in Src kinase phosphorylated at Tyr416, which was accompanied by an increase in Tyr527 phosphorylation levels. When we compare these results with those obtained in cells exposed to sorbitol but transfected with siACP1-CV, we observed an increase in Tyr416 phosphorylated Src kinase, while no significant alteration in Tyr527 phosphorylation status occurred (Fig. 5D). These results demonstrated the important role played by LMWPTP during hyperosmotic stress in the regulation of Tyr416 phosphorylation status and Src kinase activation.

**Fig 6:**
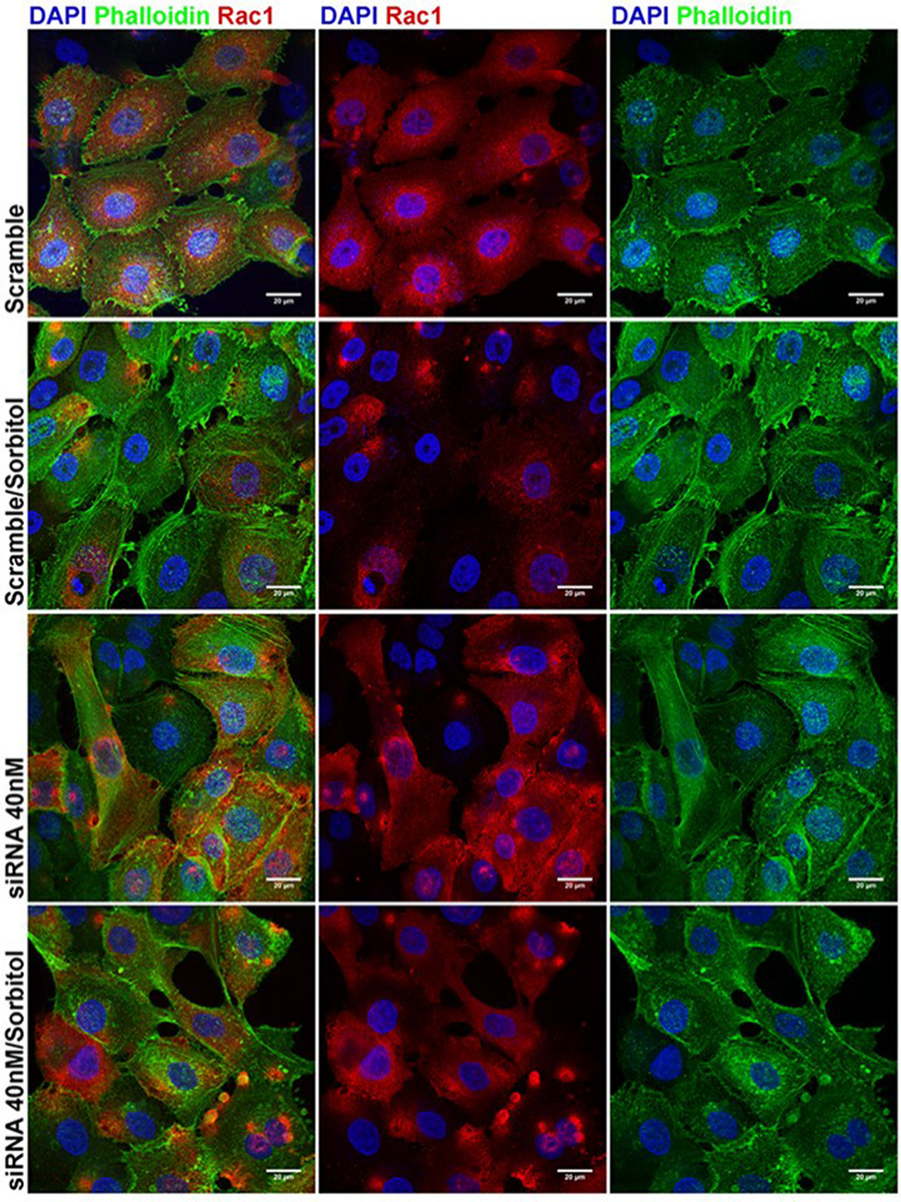
Rac-1 and F-actin staining in LMWPTP silenced HaCaT cells. HaCaT cells were transfected with siACP1-CV 40 nM and exposed to 1 M sorbitol for 2 h. Rac-1 distribution and the organization of the actin filaments (F-actin) were evaluated by laser confocal microscopy after incubation of the cells with specific antibody for Rac-1, followed by staining with Alexa Fluor 594 goat anti-rabbit IgG antibody (red), and Alexa Fluor 488-conjugated phalloidin (green). The nuclei were stained with DAPI (blue). Bar = 20 μm. Representative results of 3 independent experiments.

As demonstrated by Nimnual et al. [48], cytoskeletal rearrangements based on Rac-1 activation involve the production of reactive oxygen species (ROS) and the consequent oxidation and inhibition of LMWPTP. As mentioned before, our model of sorbitol-induced hyperosmotic stress did not cause alteration in Rac-1 expression, which is in agreement with our results showing increased activity of LMWPTP. In addition, since LMWPTP is able to dephosphorylate Rho-GAP, contributing to the maintenance of active Rho-A, it is possible to suggest that beside the demonstrated increase in Rho expression, the activity of this protein is also increased. Interestingly, our results showed that LMWPTP silencing in HaCaT cells (without exposition to sorbitol) increased Rac-1 expression, suggesting that LMWPTP also influences the expression of this small GTPase (Fig. 5A and B). The same pattern of results was obtained when expression of Rac-1 was analyzed by confocal microscopy (Fig. 6). In addition, the results also showed that when LMWPTP-siRNA transfected cells were treated with sorbitol, a lack of inhibition of Rac-1 expression was observed compared with its expression in Scramble-siRNA transfected cells (Fig. 5A and C). The same pattern of results was obtained when expression of Rac was analysed through confocal microscopy (Fig. 6). These results demonstrated that LMWPTP is involved in the mechanisms associated with Rac-1 expression and that the expression of this phosphatase is involved in the effects of sorbitol treatment in Rac-1 during hyperosmotic stress. How LMWPTP activity inhibits the expression of Rac-1 is still an unknown issue. Moreover, its effect on cytoskeleton was also suggested as these results were followed by changes in F-actin organization, with a different distribution at the leading edge (Fig. 6).

Since STAT5 is a known substrate of LMWPTP [49,50] and HaCaT cells exposed to sorbitol showed an increase in STAT5 expression and Tyr694 phosphorylation levels, a possible role of LMWPTP in STAT5 phosphorylation during sorbitol stress was evaluated in HaCaT cells. First, the results showed that in non-stressed cells LMWPTP silencing caused a marked decrease in STAT5 Tyr694 phosphorylation, indicating a positive correlation between LMWPTP activity and STAT5 activation (Fig. 5A and E). Curiously, when these cells were treated with sorbitol, the levels of STAT5 Tyr694 phosphorylation did not change significantly (Fig. 5A and E). Although phosphorylated STAT5 is known as a LMWPTP substrate, our results indicate that in the hyperosmotic stress context, LMWPTP indirectly contributes to the maintenance of STAT5 phosphorylation. It is known that STAT5 phosphorylation at Tyr694 is associated, mainly, to the activation of JAK2, although other kinases could exert the same activity, as the case of the leukemia-related BCR-ABL tyrosine kinase. Also, the reversible tyrosine phosphorylation of the STAT proteins is regulated by SH2-domain-containing protein tyrosine phosphatase-2 (SHP-2), PTP1-B, as well as serine protein phosphatase 2A (PP2A) [51]. Studies are necessary to uncover the effects of LMWPTP on the activity of such phosphatases and kinases in order to improve our knowledge on the functions of this phosphatase in hyperosmotic stress conditions.

## Discussion

Hyperosmotic stress removes water out of cell causing cell shrinkage and increasing ionic strength, which ultimately can lead to alterations in cellular architecture compartments, denaturation of proteins, and perturbation of cell functions [2,52,53]. Additionally, osmotic shock activates cytoskeleton reorganization, although less is known about the biochemical mechanisms involved in this response. In this work, exposure of HaCaT cells to sorbitol demonstrates that hyperosmotic stress induces apoptosis in keratinocytes, as previously reported by Diker-Cohen et al. [2], since a marked increase in annexin V staining, caspase-3 activation and PARP-1 cleavage were demonstrated. In addition, cytoskeleton rearrangements were also triggered by sorbitol during cell death as indicated by Src kinase inhibition, LMWPTP activation, alterations in Rho and Rac-1 expression, and changes in F-actin organization.

Several aspects of the physiological action of LMWPTP in the regulation of cellular metabolism have been described in different biological conditions. Of interest, the ability of LMWPTP to interact with Src kinase in the control of cellular differentiation and cytoskeleton rearrangement has been studied [18,20,43]. The activity of the Src kinase mediates cell adhesion and cytoskeleton rearrangements, thus affecting downstream protein activation [41,42]. Our data demonstrated that exposure of HaCaT keratinocytes to sorbitol-induced osmotic stress inhibited the Src kinase activity, as indicated by the downregulation of phospho-Tyr416 located in the activation loop of the enzyme and the increase in phospho-Tyr527, a known inhibitory site of this kinase. As mentioned before, the Src kinase pathway acts in osmotically stressed keratinocytes by inhibiting cell death [1,7], supporting the idea that its inhibition might contribute to the observed cell death. Concomitantly to Src inhibition, we reported the activation of LMWPTP, which was also demonstrated by the evaluation of phospho-Tyr levels in immunoprecipitated LMWPTP. Interestingly, LMWPTP is able to dephosphorylate Src kinase mainly at Tyr416 [18], thus explaining the reduction in Tyr416 phosphorylated form of Src kinase observed in our study. It is very important to mention that the regulation of LMWPTP is based on phosphorylation/dephosphorylation and a change in ROS concentration modulates reversible oxidation of cysteine residues.

The activity of protein kinases is finely regulated in cells since these proteins are key players in the regulation of signaling pathways. It is thought that the redox status of the intracellular milieu exerts high influence in the regulation of both protein kinases and phosphatases, leading to the activation of the former and inactivation of the later group. In this respect, our results demonstrating that Src activity was inhibited by sorbitol-induced stress, in contrast to the activation of LMWPTP, reinforce the modulation of ROS action by antioxidant mechanisms [22]. Previous work showed the importance of the glutathione/glutathione reductase/NADPH system in the reversible oxidation of LMWPTP, which is dependent on the availability of GSH [47]. In this way, our results indicate that GSH maintains the intracellular redox balance and protects the sulfenic derivative of LMWPTP from further oxidation, driving the formation of a more stable product, and creating ideal conditions for LMWPTP action in response to hyperosmotic stress. In addition to GSH, upregulation of GR and GPX expression, and raise in GST activity were also detected after treatment of HaCaT with sorbitol. The effectiveness of this antioxidant response could be observed through the reduced lipid peroxidation, inhibition of Src activity and, importantly, LMWPTP activity.

A recent report has demonstrated that LMWPTP also plays a role in modulating Src activity in osteoblast differentiation [18]. According to these authors, modest activation of LMWPTP leads to dephosphorylation of the kinase at Tyr527 and Tyr416, the inhibitory and stimulatory residues of the kinase activity, respectively. Although our results demonstrate an increase in Tyr527 phosphorylation after exposure of keratinocytes to sorbitol, a significant reduction in Tyr416 was also observed, suggesting an action of LMWPTP in such event. Why the phosphatase could not dephosphorylate Tyr527 is a theme for a further study but we can hypothesize that this event is associated with the selectivity of the enzyme towards its substrate depending on its phosphorylation status. Indeed, phosphorylation of LMWPTP in Tyr132 residue is able to regulate the selectivity of the phosphatase towards diverse substrates. Once LMWPTP is phosphorylated at Tyr132 the enzyme becomes able to interact with SH2 domain-containing molecules such as Grb2, a known adaptor protein in signaling pathways. Furthermore, as Tyr132 is located close to the active site, the interaction of Grb2-like proteins with Tyr132-phosphorylated LMWPTP could limit the access of the ligand-binding surface and active site entrance, leading to enzyme inactivation or selection of substrates by size [54]. Possibly, in our model, LMWPTP becomes able to dephosphorylate Src at Tyr416, but unable to dephosphorylate this enzyme at Tyr527.

In addition to Src, other cytoskeletal proteins were altered in keratinocytes by hyperosmotic shock. Alteration in F-actin organization and increased protein expression was found for the small GTPases RhoA and Rac-1, whereas cofilin expression was decreased and its phosphorylation at Ser3 was increased. Cofilin is an actin-binding protein that is required for actin filament disassembly and rearrangement. In physiological conditions, PP2A, a Ser/Thr phosphatase, is able to control cofilin activation by dephosphorylating its Ser3 residue [55]. Thus, our results demonstrating strong inhibition of PP2A during hyperosmotic shock is consistent with the inactivation of cofilin in keratinocytes.

Finally, the direct action of LMWPTP in the molecular events associated with keratinocyte hyperosmotic stress response was demonstrated by the effects of knocking down the ACP1 gene. Inhibition of LMWPTP expression was able to attenuate the effects of sorbitol in HaCaT cells mainly at the control of Src and STAT5 activities and Rac expression. Indeed, even in non-stressed cells LMWPTP showed to be a key player in the regulation of expression and activity of Src, FAK and Rac proteins.

## Conclusions

In summary, our work provides new contribution to the mechanisms involved in the response of keratinocytes to hyperosmotic shock, in which LMWPTP may play an important role in cytoskeleton rearrangement. To the best of our knowledge, up until now, there was no study describing a role for LMWPTP in keratinocytes upon exposure to stressful conditions.
